# Unraveling immunotherapeutic targets for endometriosis: a transcriptomic and single-cell analysis

**DOI:** 10.3389/fimmu.2023.1288263

**Published:** 2023-11-16

**Authors:** Cankun Zhou, Minqing Feng, Yonglian Chen, Side Lv, Yifan Zhang, Jiebo Chen, Rujian Zhang, Xiaobin Huang

**Affiliations:** ^1^ Department of Gynecology, Southern Medical University Affiliated Maternal & Child Health Hospital of Foshan, Foshan, Guangdong, China; ^2^ Southern Medical University, Graduate School, Guangzhou, Guangdong, China; ^3^ Department of Gynecology, Shenzhen People’s Hospital, Shenzhen, Guangdong, China

**Keywords:** endometriosis, single-cell RNA sequencing, CIBERSORT, immune, molecular docking

## Abstract

**Background:**

Endometriosis (EMs), a common gynecological disorder, adversely affects the quality of life of females. The pathogenesis of EMs has not been elucidated and the diagnostic methods for EMs have limitations. This study aimed to identify potential molecular biomarkers for the diagnosis and treatment of EMs.

**Methods:**

Differential gene expression (DEG) and functional enrichment analyses were performed using the R language. WGCNA, Random Forest, SVM-REF and LASSO methods were used to identify core immune genes. The CIBERSORT algorithm was then used to analyse the differences in immune cell infiltration and to explore the correlation between immune cells and core genes. In addition, the extent of immune cell infiltration and the expression of immune core genes were investigated using single-cell RNA (scRNA) sequencing data. Finally, we performed molecular docking of three core genes with dienogest and goserelin to screen for potential drug targets.

**Results:**

DEGs enriched in immune response, angiogenesis and estrogen processes. CXCL12, ROBO3 and SCG2 were identified as core immune genes. RT-PCR confirmed that the expression of CXCL12 and SCG2 was significantly upregulated in 12Z cells compared to hESCs cells. ROC curves showed high diagnostic value for these genes. Abnormal immune cell distribution, particularly increased macrophages, was observed in endometriosis. CXCL12, ROBO3 and SCG2 correlated with immune cell levels. Molecular docking suggested their potential as drug targets.

**Conclusion:**

This study investigated the correlation between EMs and the immune system and identified potential immune-related biomarkers. These findings provided valuable insights for developing clinically relevant diagnostic and therapeutic strategies for EMs.

## Introduction

Endometriosis (EMs), which is one of the prevalent gynecological disorderrs among women of reproductive age, is characterized by the occurrence of endometrial tissues (glands and stroma) outside the uterine cavity. Globally, EMs affects approximately 10% of women in their reproductive years with the highest incidence observed among women aged 25–35 years (1). Approximately 190 million women suffer from EMs worldwide (2). The prevalence rates of EMs in the asymptomatic, infertile, and pelvic pain groups are 2%–11%, 5%–50%, and 5%–21%, respectively. In symptomatic adolescents, the prevalence rate of EMs is in the range of 49%–75% (3). The symptoms of EMs include pain, development of masses and nodules, and infertility, which adversely affect the quality of life of females. The incidence of co-morbidities in females with EMs is higher than that in females without EMs. The infertility incidence rate is as high as 40%–50% in patients with EMs (2). The risk of early recurrent spontaneous abortion in pregnant women with EMs is significantly higher than that in healthy pregnant women (4).Furthermore, patients with EMs are at an increased risk of developing clear cell and endometrioid ovarian cancer, non-Hodgkin’s lymphoma, thyroid cancer, and endometrial cancer (3, 5, 6). Treatments for EMs include surgery, drug therapy, and assisted reproductive technologies. However, these treatments are not effective in some patients. In cases where the treatments are effective, approximately 50% of patients experience recurrence within five years of treatment (7) The average time lag between the onset of symptoms and the definitive diagnosis of EMs is 6–7 years (8). Currently used diagnostic methods for EMs are associated with advantages and disadvantages. Thus, there is a need to develop a combined diagnostic approach to reduce the delay and increase the precision of EMs diagnosis. Additionally, preventive strategies, as well as therapeutic strategies, for EMs must be developed to alleviate the suffering of patients. Furthermore, the molecular biomarkers for the diagnosis and treatment of EMs must be identified.

Several studies have examined the etiology of EMs although it has not been completely elucidated. Various hypotheses have been proposed for the underlying mechanisms and pathological determinants of EMs. In particular, the pivotal role of immune factors in EMs development has been the recent research focus (9–11). The occurrence of EMs is directly influenced by dysfunctional immune mechanisms and related factors (12, 13). Patients with EMs exhibit alterations in the peritoneal immune microenvironment and are characterized by augmented peritoneal fluid volume, compromised phagocytic function of peritoneal macrophages, reduced cytotoxicity of natural killer (NK) cells, variations in the quantity and functional properties of T lymphocytes and B lymphocytes, and upregulated levels of pro-inflammatory and chemotactic cytokines originating from macrophages and other cellular sources (9). This aberrant peritoneal immune environment can facilitate the immune escape of ectopic endometrial tissue, providing immune conditions for the implantation, invasion, and infiltration of endometriotic lesions and consequently inducing the development of EMs (14). Limited studies have examined the mechanisms underlying this process, emphasizing the need for further research in this domain. The elucidation of the correlation between immunological dysregulation and EMs will enable the identification of novel therapeutic targets and diagnostic modalities and contribute to alleviating the suffering of patients with EMs.

In this study, the microarray datasets were retrieved from the Gene Expression Omnibus (GEO) database (15) (GSE141549) and analyzed using R language. EMs was closely associated with pathways related to the immune system (including Antigen processing and presentation, T helper cell 17 (Th17 cell) differentiation, Th1 and Th2 cell differentiation, Leukocyte transendothelial migration), immune disorders (Rheumatoid arthritis and Systemic lupus erythematosus), and drug metabolism-cytochrome P450. Additionally, three potential immune-related biomarkers (C-X-C Motif Chemokine Ligand 12 (CXCL12), Secretogranin II (SCG2), Roundabout Guidance Receptor 3 (ROBO3)) for EMs were identified and a diagnostic model based on these biomarkers was developed. The expression levels CXCL12, SCG2, and ROBO3 were validated at the cellular level. Furthermore, the cell-type identification by estimating relative subsets of RNA transcripts (CIBERSORT) algorithm and single-cell RNA sequencing (scRNA-seq) analyses were performed to examine the correlation between these biomarkers and immune cell infiltration. The findings of this study provided valuable insights for the development of diagnostic and therapeutic strategies for EMs.

## Materials and methods

### Data collection and processing

The GSE141549 (16), and GSE7305 (17) datasets were downloaded from the GEO database (https://www.ncbi.nlm.nih.gov/geo/) and used as the test cohort and validation datasets, respectively (**Table 1**). Next, scRNA-seq data (GSE179640 (18)), comprising the data of 9 eutopic endometrium samples (GSM6102537, GSM6102540, GSM6102543, GSM6102546, GSM6102549, GSM6102551, GSM6102554, GSM6102555, and GSM6102560) and 12 EMs samples (GSM6102536, GSM6102539, GSM6102542, GSM6102545, GSM6102548, GSM6102550, GSM6102552, GSM6102553, GSM6102556, GSM6102559, GSM6102561, and GSM6102562) were downloaded. The raw data from the GSE141549 dataset were processed using the “limma” package (19) in the R environment to standardize the data and remove the data of genes that were missing, duplicated, and highly downregulated. differential expression analysis was performed to identify the differentially expressed genes (DEGs) between the EMs and control groups. Volcano plots and heatmaps were used to visually depict the distribution and expression of DEGs. The DEGs were identified based on the following criteria: an absolute fold change greater than 1 (|log2FC|>1) and an adjusted p-value less than 0.05 (adj.P.Val<0.05). Next, the DEGs were subjected to functional enrichment analysis using the Gene Ontology (GO) and Kyoto Encyclopedia of Genes and Genomes (KEGG) (20) modules of the OmicShare tool (https://www.omicshare.com/tools/).

**Table 1:** GEO data collection table.

Datasets	Accession	Platform	Endometriosis	Endometrium
Microarray	GSE141549	GPL13376	79	61
	GSE7305	GSE7305	10	10

### Screening of the critical immune genes

The immune gene set was sourced from the IMMPORT website (http://www.immport.org/, **Supplementary File 1**). A scale-free weighted gene co-expression network of the GSE141549 dataset was constructed using the “Weighted Gene Coexpression Network Analysis (WGCNA)” toolkit (21) in R to identify co-expressed genes and modules associated with EMs. The intersection of the gene set within the module exhibiting the closest association with EMs, the DEGs, and the immune gene set facilitated the identification of key immune genes.

### Key immune gene screening and diagnostic model construction

Random forest (RF) model, support vector machine-recursive feature elimination (SVM-RFE) algorithm, and least absolute shrinkage and selection operator regression (LASSO) regression were used to analyze the critical immune genes and screen the corresponding diagnostic genes. Key immune genes associated with EMs were identified using the intersection set. To examine the diagnostic efficacy of these genes, we calculated the area under the receiver operating characteristic (ROC) curve (AUC) for each key gene was calculated. The expression and diagnostic potential of these genes were validated using the GSE7305 datasets. Additionally, we conducted gene set enrichment analysis (GSEA) was performed to evaluate the potential functional roles of these genes in the context of EMs. Finally, a diagnostic column line graph was generated using the “Rms” package (22) in R to predict the occurrence of EMs. The consistency index (C-index) and calibration curve set decision curve analysis (DCA) were used to assess the predictive power and clinical application of the model.

### Immune infiltration analysis

The CIBERSORT algorithm (23) was used to investigate differential immune cell infiltration between the EMs and control groups. Additionally, the correlation between immune cells and the key genes was examined using Spearman’s rank correlation coefficient.

### scRNA-seq analysis

The “Seurat” package in R was used to analyze the scRNA-seq data. The data of low-quality cells, which were determined based on the following criteria, were censored from the analysis: cells expressing less than 200 genes and those expressing less than 3 genes; cells with < 1000 genes; cells with < 200 or > 10000 Unique Molecular Identifiers (UMIs) <200 or >10000; mitochondrial genes expressed in more than 25% of cells and ribosomal genes expressed in more than 20% of cells. Next, the “NormalizeData” function in Seurat was used to perform data normalization, followed by batch correction and dimensionality reduction using Harmony. The top 2000 highly variable genes were identified. Cell clusters were identified using 20 principal components (PCs) at resolution of 0.7, resulting in the subgrouping of all cells into 28 clusters. The DEGs for each cluster were identified using the “FindAllMarkers” function in Seurat with the following parameters: “min.pct = 0.25” and “logfc.threshold = 0.25”.

Single-cell annotation was performed using singleR. For accurate annotation, manual correction was performed, referencing established markers from published literature. The following marker genes were used for annotation: Platelet Derived Growth Factor Receptor Beta (PDGFRB) for (epithelial cells marker), Platelet And Endothelial Cell Adhesion Molecule 1 (PECAM1) and CD34 Molecule (CD34) for (endothelial cells marker), Collagen Type I Alpha 1 Chain (COL1A1), Collagen Type III Alpha 1 Chain (COL3A1), Collagen Type I Alpha 2 Chain (COL1A2), Fibroblast Growth Factor 7 (FGF7),and Membrane Metalloendopeptidasee (MME) for (fibroblasts markers), CD79a Molecule (CD68), CD19 Molecule (CD19), and Membrane Spanning 4-Domains A1 (MS4A1) for (B cells markers), CD3d Molecule (CD3D), CD3e Molecule (CD3E), and CD3g Molecule (CD3G) for (T cells markers), T Cell Receptor Delta Constant (TRDC), Killer Cell Lectin Like Receptor C1 (KLRC1), Killer Cell Lectin Like Receptor D1 (KLRD1), Natural Killer Cell Granule Protein 7 (NKG7), Killer Cell Lectin Like Receptor B1 (KLRB1), Neural Cell Adhesion Molecule 1 (NCAM1/CD56), and Killer Cell Lectin Like Receptor G1 (KLRG1) for (NK cells markers), CD14 Molecule (CD14) for Monocytes, CD68 Molecule (CD68) for (M1 macrophages marker), Colony Stimulating Factor 1 Receptor (CSF1R/CD115), CD163 Molecule (CD163), Macrophage Scavenger Receptor 1 (MSR1/CD204), and Mannose Receptor C-Type 1 (MRC1/CD206) for (M2 macrophages marker), Integrin Subunit Alpha X (ITGAX/CD11C) and CD1c Molecule (CD1C) for (dendritic cells marker), Membrane Spanning 4-Domains A2 (MS4A2), Glycoprotein IX Platelet (GP9/CD42a), and Glycoprotein Ib Platelet Subunit Alpha (GP1BA/CD42B) for (mast cells marker). Finally, a histogram of the content ratio of each cell, the distribution of EMs-related key immune genes expressed in single cells and gene differences was plotted.

### Protein-ligand interaction analysis

The three-dimensional structure images of the three core target proteins were obtained from Uniprot (24) (https://www.uniprot.org/). The structures of dienogest and goserelin were downloaded from PubChem (https://pubchem.ncbi.nlm.nih.gov/) in the structure data file (SDF) format. Then OpenBabel software was used to transform the obtained SDF structures into mol2 structures. The protein receptors and small molecule ligands were imported into AutoDock software for molecular docking. The docking results were plotted using PyMol software.Binding pairs with binding energy values of < −1.2 kcal/mol were considered to exhibit good binding. The binding energy values were inversely proportional to the docking ability.

### Quantitative real-time polymerase chain reaction

The eutopic stromal cell line (human embryonic stem cells (hESCs)) and the immortalized endometriotic epithelial cell line (12Z cells) were procured from ABM (Richmond, BC, Canada). Cells were cultured in Dulbecco’s modified Eagle’s medium/F12 (DMEM/F12; Gibco) supplemented with 10% fetal bovine serum (FBS; Gibco) and 1% penicillin-streptomycin (PS; Gibco) at 37°C and 5% CO_2_ in an incubator. Total RNA was extracted from hESCs and 12Z cells using the cell total RNA isolation kit (Foregene) and RNA extraction kits, following the manufacturer’s instructions. The RNA samples were subjected to reverse transcription using the 5× PrimeScript RT Master Mix (Takara). The resulting complementary DNA was quantified using the TB Green^®^ Premix Ex Taq™ II (Takara) with the CFX96 touch q-PCR system (Bio-Rad Laboratories,Inc.). The relative expression levels of the target gene were determined using the 2^−ΔΔCt^. ACTB served as the internal reference. The experiments were repeated thrice for robustness. The primer sequences used in qRT-PCR analysis are shown in **Supplementary Table 1**.

### Statistical analysis

All statistical analyses were performed using R software (version 4.1.2). The differential core genes expression and immune cell infiltration levels were compared using the Wilcoxon test. Spearman analysis was performed to evaluate the correlation analysis. Differences were considered significant at p < 0.05.

## Results

### DEG screening and functional enrichment analysis

**Figure 1:**
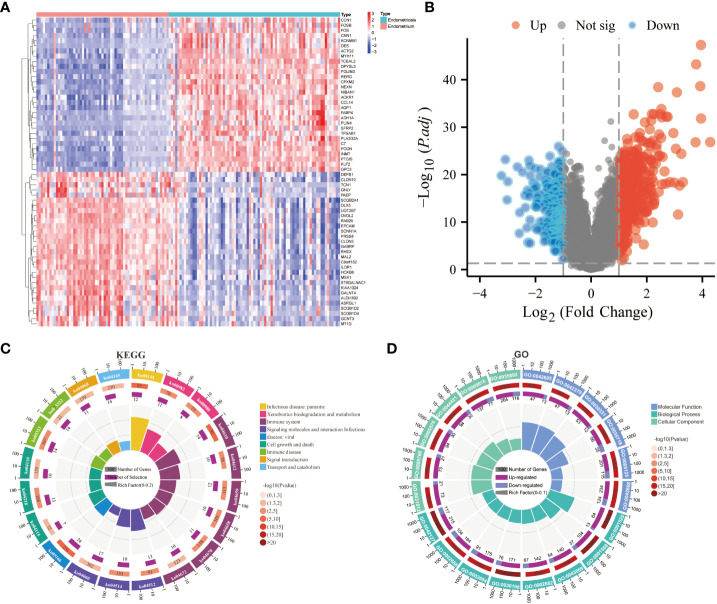
Screening of differentially expressed genes (DEGs) and functional enrichment analysis. **(A)** Heatmap of DEGs. **(B)** Volcano plot of DEGs. **(C)** Kyoto Encyclopedia of Genes and Genomes (KEGG) enrichment analysis of DEGs. **(D)** Gene Ontology (GO) enrichment analysis of DEGs.

After the standardization of the microarray data (GSE141549), 647 DEGs(247 downregulated genes and 399 upregulated genes) were identified using the “limma” package (**Supplementary File 2**) (**Figures 1A, B**). The biological functions of these DEGs were examined using GO annotation and KEGG pathway enrichment analyses. The DEGs were significantly enriched in immune-related processes, encompassing antigen processing and presentation, Th17 cell differentiation, Th1 and Th2 cell differentiation, and leukocyte transendothelial migration. Furthermore, the DEGs were associated with the pathways related to immune diseases (such as rheumatoid arthritis and systemic lupus erythematosus) and drug metabolism-cytochrome P450 (**Figure 1C**; **Supplementary Table 2**). The DEGs were enriched in different GO terms as follows: biological process terms, noteworthy enrichments were observed in critical processes, including blood vessel development, angiogenesis, regulation of the immune system process, regulation of leukocyte chemotaxis, and positive regulation of the immune system process; molecular function terms, chemokine receptor binding, cytokine activity, T cell receptor binding, estrogen receptor binding, and macrophage migration inhibitory factor binding; cellular component terms, extracellular structures and pathways related to macrophage migration (**Figure 1D**; **Supplementary Table 3**). The results of KEGG and GO revealed that EMs is associated with immune responses, vascular development, and estrogen-related processes.

### Weighted gene co-expression network construction

**Figure 2:**
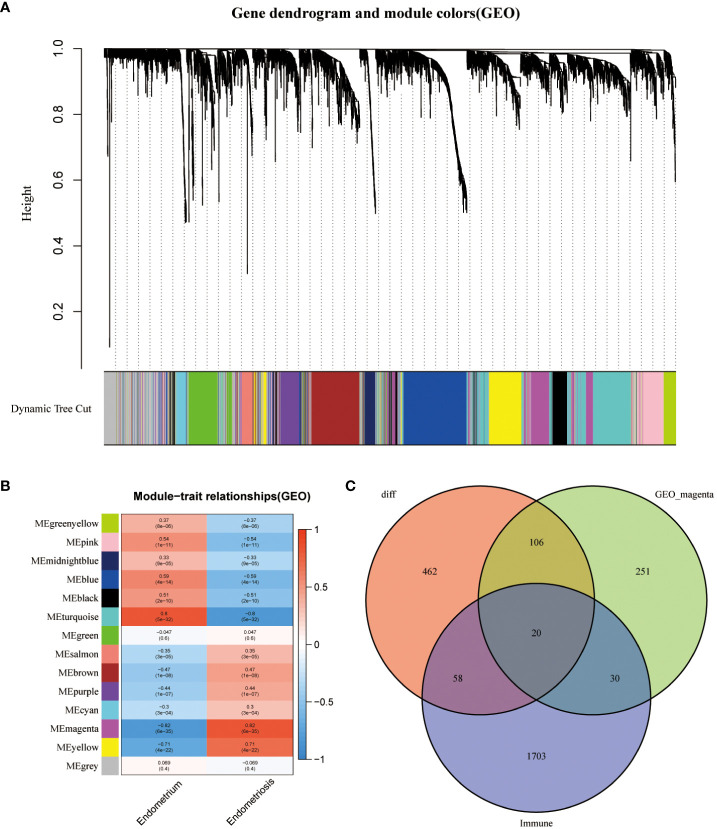
Construction of weighted gene co-expression (WGCNA) network. **(A)** Screening the co -expression module. **(B)** Heatmap of module-trait correlations (red and blue indicate positive and negative correlations, respectively). **(C)** Venn plot of key module genes versus DEGs and immune genes.

The data of samples in the GSE141549 database were clustered, and the soft threshold was set to 9 (**Supplementary Figure 1B**). A co-expression network was established when the R2 value was > 0.9, indicating high average connectivity (**Figure 2A**). Among the 14 gene modules identified, the MEmagenta module showed the highest correlation (0.82) with EMs (**Figure 2B**). The MEmagenta module comprised 407 genes (**Supplementary File 3**). The intersection of genes within the MEmagenta module DEGs, and immune-related genes revealed the following 20 critical immune genes associated with EMs: Bone Marrow Stromal Cell Antigen 2 (BST2), C-C Motif Chemokine Ligand 19 (CCL19), C-C Motif Chemokine Ligand 21 (CCL21), CXCL12, Fatty Acid Binding Protein 4 (FABP4), Growth Hormone Receptor (GHR), LIM Zinc Finger Domain Containing 1 (LIMS1), LDL Receptor Related Protein 1 (LRP1), Latent Transforming Growth Factor Beta Binding Protein 2 (LTBP2), Nerve Growth Factor (NGF), Platelet Derived Growth Factor Receptor Like (PDGFRL), Phospholipase A2 Group IIA (PLA2G2A), Prostaglandin F Receptor (PTGFR), ROBO3, Sphingosine-1-Phosphate Receptor 1 (S1PR1), SCG2, Semaphorin 3C (SEMA3C), Transforming Growth Factor Beta 3 (TGFB3), Transforming Growth Factor Beta Receptor 2 (TGFBR2), and Wnt Family Member 5A (WNT5A) (**Figure 2C**).

### Selection and validation of three core immune genes (CXCL12, ROBO3, and SCG2)

**Figure 3:**
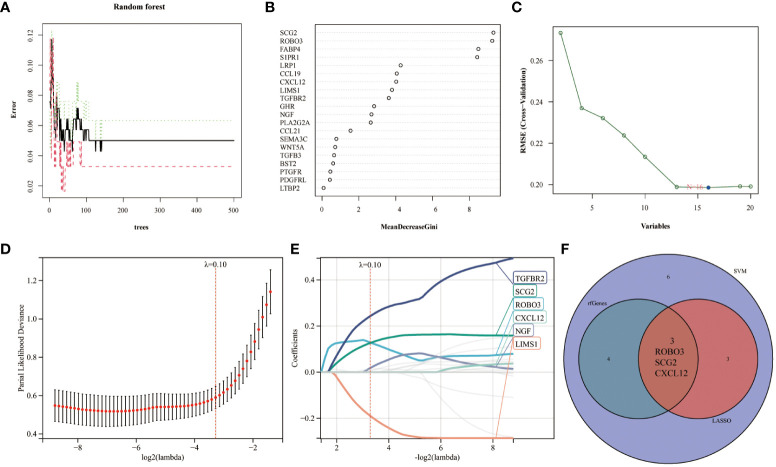
Multiple machine learning models were used to identify core immune genes (CXCL12, ROBO3, and SCG2) in the GSE141549 dataset. **(A, B)** The genes were selected based on the random forest (RF) model. **(C)** The support vector machine (SVM) algorithm was used to select the genes. **(D, E)** Least absolute shrinkage and selection operator (LASSO) regression analysis was used to select the genes. **(F)** Three key immune genes identified from the Venn diagram.

Immunological biomarkers with diagnostic significance were identified using three machine learning algorithms. The RF model identified seven genes (SCG2, ROBO3, FABP4, S1PR1, LRP1, CCL19, and CXCL12) based on the following criterion: importance scores > 4 (**Figures 3A, B**). Next, the SVM-RFE algorithm identified 16 genes (SCG2, ROBO3, S1PR1, FABP4, NGF, LIMS1, PLA2G2A, LRP1, TGFBR2, CCL19, PDGFRL, CXCL12, GHR, PTGFR, CCL21, and TGFB3) (**Figure 3C**). Finally, the LASSO regression analysis yielded six genes (TGFBR2, SCG2, ROBO3, CXCL12, NGF, and LIMS1) from the optimal model (**Figures 3D, E**). The intersection of these genes using a Venn diagram revealed three robust core biomarkers (CXCL12, ROBO3, and SCG2) (**Figure 3F**).

**Figure 4:**
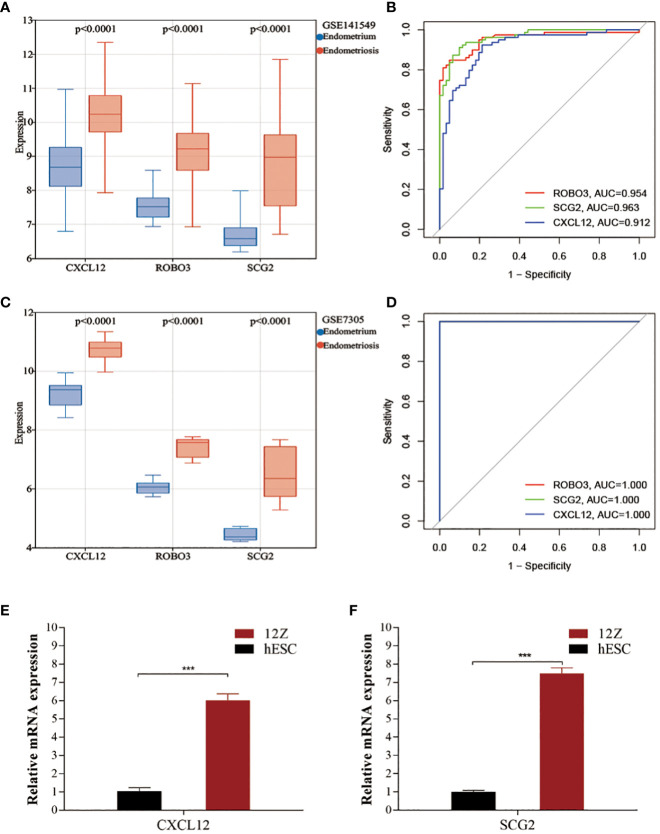
Validating the expression and diagnostic value of core immune genes. **(A, C)** The expression levels of CXCL12, ROBO3, and SCG2 in the test (GSE141549) and validation cohorts (GSE7305). **(B, D)** Receiver operating characteristic (ROC) curves for evaluating the diagnostic values of CXCL12, ROBO3, and SCG2 in the test (GSE141549) and validation cohorts (GSE7305). **(E, F)** The expression levels of CXCL12 and SCG2 in 12Z cells and hESCs cells.

Compared with those in the endometrium samples, the CXCL12, ROBO3, and SCG2 levels were significantly upregulated in the EMs samples (**Figure 4A**). The expression patterns of these three genes inEMs were validated using the GSE7305 dataset. Consistently, the CXCL12, ROBO3, and SCG2 were significantly upregulated in the EMs samples in the GSE7305 dataset (**Figure 4C**). qRT-PCR analysis was performed to further validate the dysregulation of CXCL12, ROBO3, and SCG2 EMs. The CXCL12 and SCG2 levels in 12Z cells were significantly upregulated when compared with those in hESCs (**Figures 4E, F**). Meanwhile, ROBO3 was downregulated in both 12Z cells and hESCs to an undetectable level. Furthermore, the diagnostic value of these genes was evaluated using ROC curve analysis. The AUC values for CXCL12, ROBO3, and SCG2 were almost all > 0.9 (**Figures 4B, D**). These findings indicate that CXCL12, ROBO3, and SCG2 are potential novel diagnostic biomarkers for EMs.

The pathways in which the three core immune genes were enriched in GSEA were similar to those in which DEGs were enriched (**Figures 1C, D**). These pathways included systemic lupus erythematosus, steroid hormone biosynthesis, leukocyte transendothelial migration, drug metabolism other enzymes, cytokine cytokine receptor interaction, and the Notch signaling pthway (**Supplementary Figures 2A–C**). Thus, CXCL12, ROBO3, and SCG2 are involved in essential immune responses and processes related to estrogen metabolism. In the validation set GSE7305, CXCL12, ROBO3, and SCG2 were significantly enriched in immune-related pathways, including cytokine-cytokine receptor interaction, chemokine signaling pathway, leukocyte transendothelial migration, systemic lupus erythematosus, and MAPK signaling pathway (**Supplementary Figures 2D–F**).

### Establishment and testing of a diagnostic column line graph

**Figure 5:**
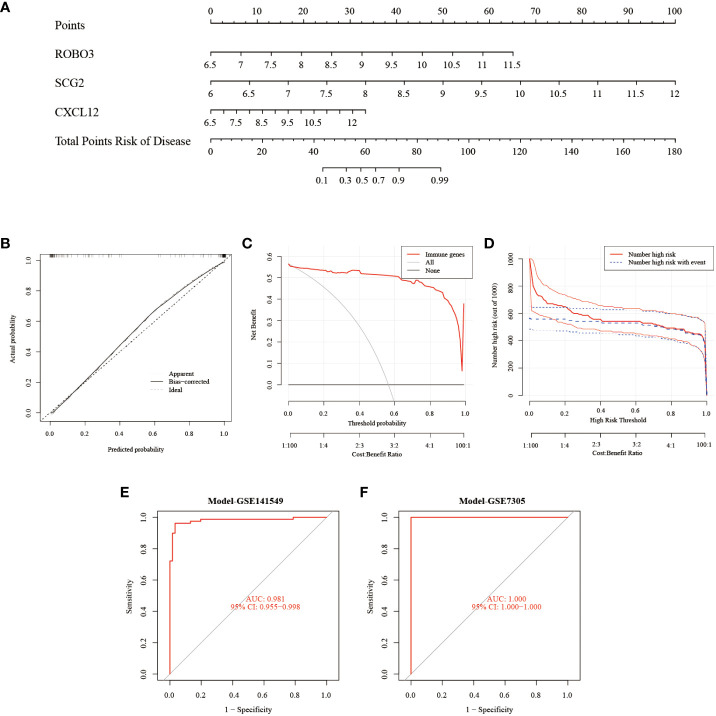
Construction and validation of the endometriosis diagnostic column line graph. **(A)** Diagnostic column line graph was used to predict the occurrence of endometriosis. **(B)** Calibration curve to examine the predictive power of the diagnostic column line graph. **(C)** Decision curve analysis (DCA) to evaluate the predictive power of the diagnostic column line graph. **(D)** Clinical impact curve to assess the predictive power of the diagnostic column line graph. **(E, F)** Receiver operating characteristic (ROC) curves to assess the clinical value of the diagnostic column line graph in the test (GSE141549) and validation cohorts (GSE7305).

The Rms package was used to construct an EMs diagnostic column line graph (**Figure 5A**). The calibration curve revealed minimal deviation between the actual event risk and the predicted risk, indicating the high accuracy of the model (**Figure 5B**). Furthermore, DCA revealed that the clinical net benefit of the diagnostic column line graph was higher than that of all other strategies (**Figure 5C**). As the high-risk threshold increased from 0.4 to 1, the “Number high risk” curve gradually overlapped with the “Number high risk with event” curve (**Figure 5D**). Additionally, the diagnostic column line graph increased high AUC values in the test cohort (GSE141549), and validation cohort (GSE7305) (0.981, 1.000, respectively; **Figures 5E, F**). These results validate the enhanced predictive performance of the diagnostic column line graph.

### Immune cell infiltration analysis

**Figure 6:**
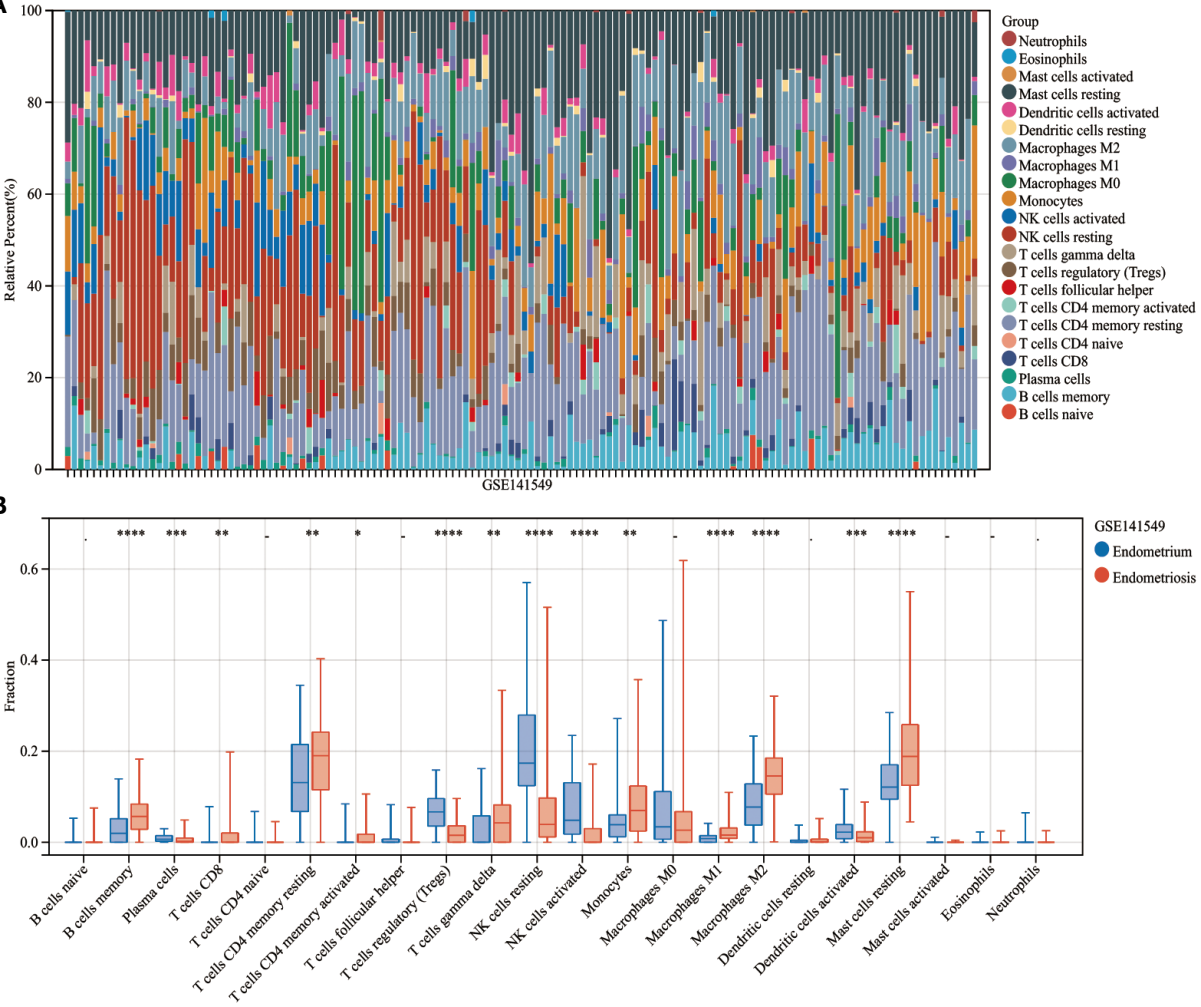
Analysis of immune landscape related to endometriosis. Heatmap **(A)** and violin plot **(B)** showing the distribution of 22 immune cells in endometrium samples and endometriosis samples of the GSE141549 dataset. *P < 0.05; **P < 0.01; ***P < 0.001;****P < 0.0001; -, non-significant (P > 0.05).

**Figure 7:**
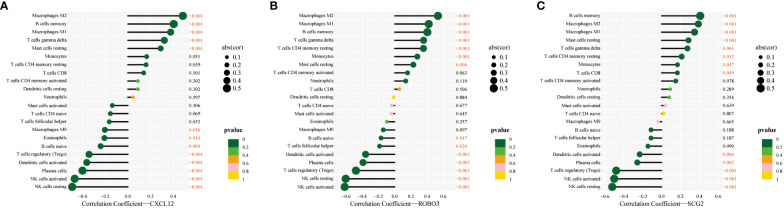
Correlation between infiltrating immune cells and core immune genes (CXCL12 **(A)**, ROBO3 **(B)**, and SCG2 **(C)**).

The CIBERSORT algorithm was used to estimate the infiltration abundance of 22 immune cell types in the samples of the GSE141549 dataset (**Figure 6A**). Comparative analysis revealed that the infiltration abundance of B cells memory, CD8+ T cells, resting CD4+ T cells memory, activated CD4+ T cells memory, T cells gamma delta, monocytes, M1 macrophages, M2 macrophages, and resting mast cells in the EMs samples were upregulated when compared with those in the endometrium tissue samples. In contrast, the infiltration abundances of plasma cells, regulatory T cells (Tregs), resting NK cells, activated NK cells, and activated dendritic cells were downregulated in the EMs samples (P<0.05, **Figure 6B**). The expression levels of CXCL12, ROBO3, and SCG2 were significantly and positively correlated with the abundances of B cells memory, M1 macrophages, M2 macrophages, resting mast cells, and T cells gamma delta. Conversely, the expression levels of CXCL12, ROBO3, and SCG2 were negatively correlated with the abundances of activated dendritic cells, activated NK cells, resting NK cells, plasma cells, and Tregs (**Figure 7**). These findings provided valuable insights into the correlation between immune cell infiltration and the expression patterns of CXCL12, ROBO3, and SCG2 in the context of EMs.

### scRNA profiling in EMs

**Figure 8:**
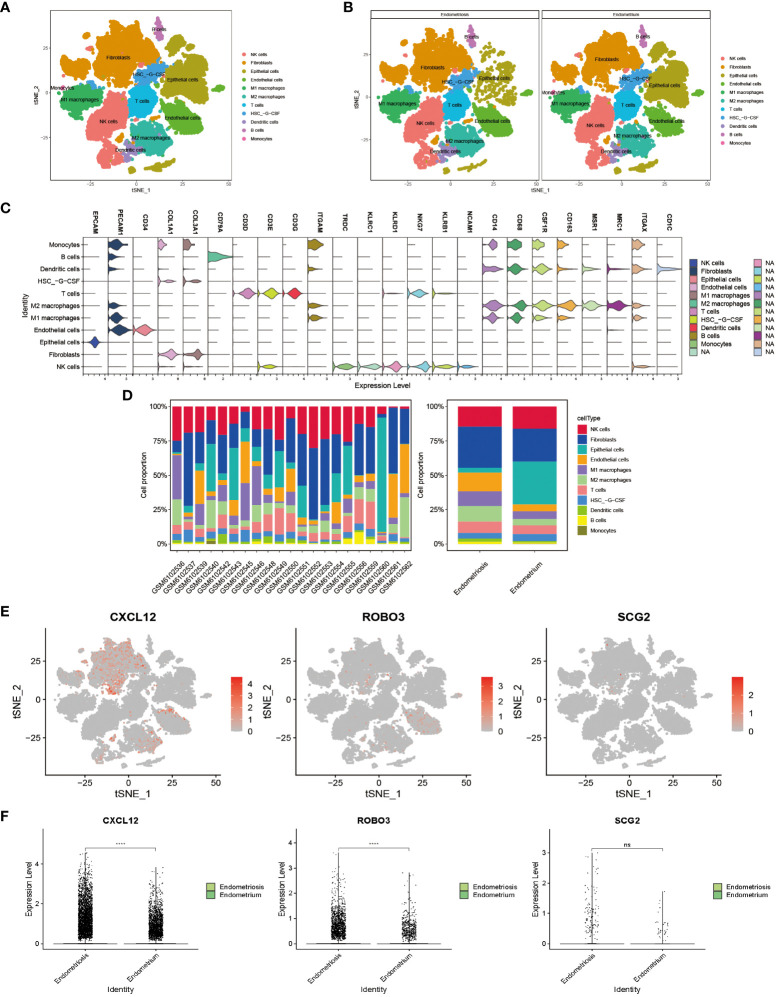
Visualization plots of single-cell RNA sequencing (scRNA-seq) data of endometriosis samples. t-Distributed stochastic neighbor embedding (tSNE) plots showing integrated analysis of eutopic endometrium and endometriosis. Cells were colored according to cell types **(A)** or tissue types **(B)**. **(C)** Violin plot of marker genes in different cell type. **(D)** Proportions of different T cell subtypes in each sample (left) or different tissue types (right). **(E)** The expression pattern of CXCL12, ROBO3, and SCG2 were representedd in the t-SNE plot. **(F)** The gene expression levels of CXCL12, ROBO3, and SCG2 in the scRNA-seq dataset (GSE179640). ****P < 0.0001; ns, non-significant (P > 0.05).

The scRNA-seq of tissues obtained from subjects with EMs and eutopic endometrium, encompassing a total of 102,095 cells before filtering (60,663 cells from patients with EMs and 41,432 cells from controls (eutopic endometrium)), were analyzed. After stringent data quality control measures, a total of 36,881 cells were retained (18,206 cells from patients with EMs and 18,675 cells from controls) (**Supplementary Figure 3A**). The data were subjected to t-distributed stochastic neighbor embedding (t-SNE) analysis. The clusters were annotated using single-cell singleR annotation. To ensure accuracy, manual annotation correction based on published markers was performed (**Supplementary Figure 3F**). Various cell types, including fibroblasts, epithelial cells, endothelial cells, T cells, B cells, NK cells, dendritic cells, monocytes, M1 macrophages, M2 macrophages, and dematopoietic stem cell_-granulocyte colony-stimulating factor (HSC_-G-CSF) (**Figures 8A–C**). The proportions of different cell subtypes in each sample and tissue type are shown in **Figure 8D** and **Table 2**. Compared with those in the endometrium group, the proportions of M1 macrophages (10.65% vs. 5.64%), M2 macrophages (11.31% vs. 4.56%), B cells (1.55% vs. 0.81%), T cells (8.29% vs. 6.38%), and dendritic cells (2.41% vs. 1.15%)) were upregulated and the proportions of NK cells (14.64% vs. 16.05%) were downregulated in the EMs group. These results are consistent with those of immune cell infiltration analysis. Next, this study focused on the distribution of core genes (CXCL12, ROBO3, and SCG2) in t-SNE plots. Based on the t-SNE plot, CXCL12 and ROBO3 were predominantly expressed in the fibroblasts, epithelial cells, and M2 macrophage subclusters (**Figure 8E**). Furthermore, the expression levels of CXCL12, ROBO3, and SCG2 were upregulated in the EMs group, with CXCL12 and ROBO3 exhibiting significant differential expression (**Figure 8F**).

**Table 2:** Cell proportion of each cell types.

Cell types	Endometrium(%)	Endometriosis(%)
Epithelial cells	31.15	3.54
Endothelial cells	5.00	13.63
Fibroblasts	24.05	29.92
B cells	0.81	1.55
T cells	6.38	8.29
NK cells	16.05	14.64
Monocytes	0.19	0
M1 macrophages	5.64	10.65
M2 macrophages	4.56	11.31
Dendritic cells	1.15	2.41

### Molecular docking of three core immune genes

**Figure 9:**
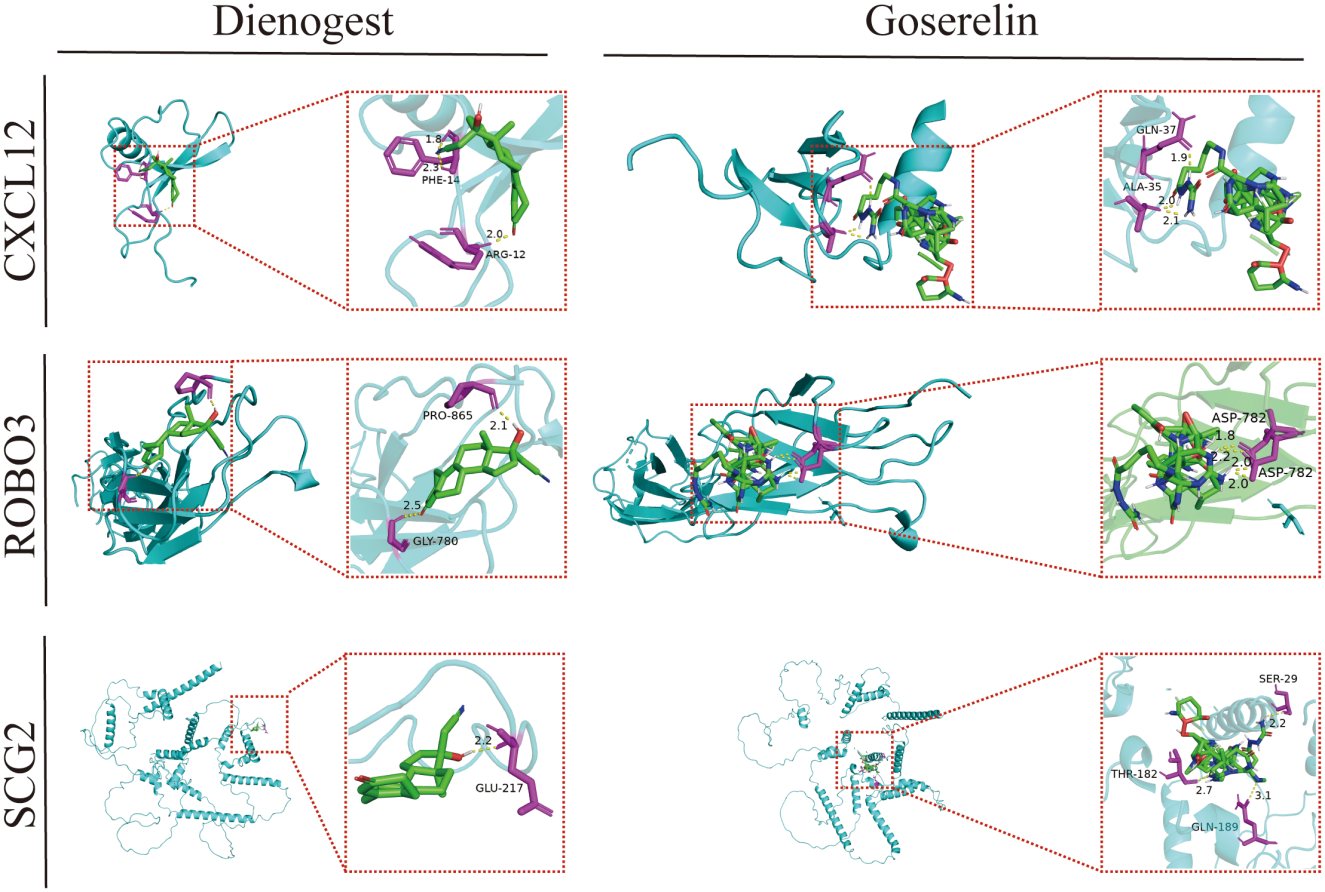
Molecular docking patterns of dienogest or goserelin with target proteins. ALA, alanine; ASP, asparticacid; ARG, arginine; GLN, glutarnine; GLU, glutamicacid; GLY, glycine; PHE, phenylalanine; RPO, proline; SER, serine; THR, threonine.

Dienogest and goserelin have been used for the treatment of EMs in several countries and regions worldwide. Molecular docking assesses the binding ability of the molecules to the target in terms of binding energy. The binding energy value was inversely proportional to the stability of the binding conformation. The three core genes could bind well to dienogest and goserelin, indicating that they have a favorable affinity (**Figure 9**; **Supplementary Table 4**).

## Discussion

The societal costs of EMs, which adversely affects the quality of life of women and their families, are similar to those of chronic diseases, such as diabetes and rheumatoid arthritis (25). Although the exact mechanisms underlying EMs development have not been elucidated, the development of EMs is hypothesized to involve the interaction between multiple factors, especially the immune components (26). The understanding of these mechanisms is limited. Hence, early detection and intervention are critical for enhancing the quality of life of patients with EMs. Additionally, the diagnosis and treatment of EMs can be further improved by identifying early diagnostic biomarkers.

This study identified 647 DEGs in EMs. Functional enrichment analysis revealed that the DEGs were enriched in pathways related to immunity, angiogenesis, and estrogen. Leveraging WGCNA and various machine learning algorithms were used to identify three core immune-related diagnostic biomarkers (CXCL12, ROBO3, and SCG2). The roles of these core immune genes in EMs were examined using GSEA. The results of GSEA were consistent with those of enrichment analysis of DEGs, which revealed the enrichment of pathways related to the immune system (such as leukocyte transendothelial migration), immune disorders (such as systemic lupus erythematosus), and estrogen metabolism (such as steroid hormone biosynthesis, drug metabolism other enzymes).

These findings indicated the critical role of the immune system. Thus, the CIBERSORT algorithm was used to comprehensively analyze the infiltration levels of 22 immune cell types. Compared with those in the healthy endometrium tissue samples to Endometriosis samples, the infiltration B cells memory, CD8+ T cells, resting CD4+ T cells memory, activated CD4+ T cells memory, T cells gamma delta, monocytes, M1 macrophages, M2 macrophages, and resting mast cells were significantly upregulated and the infiltration levels of plasma cells, Tregs, resting NK cells, activated NK cells, and activated dendritic cells downregulated in the EMs samples. scRNA sequencing data analysis revealed 11 distinct cell type clusters. The levels of these 11 clusters varied between the endometrium group and the EMs group. Compared with those in the endometrium group, the proportions of M1 macrophages, M2 macrophages, B cells, T cells, and dendritic cells were upregulated and the proportions of NK cells were downregulated in the EMs group. Furthermore, the expression levels of CXCL12, ROBO3, and SCG2 were correlated with the infiltration levels of multiple immune cells. These findings indicate that CXCL12, ROBO3, and SCG2 are potential therapeutic targets for EMs.

Genetic and environmental factors are key factors involved in the development of EMs. In particular, immune dysfunction is involved during the entire pathological process of EMs (2, 27). Suryawanshi et al. (28) reported he presence of an immune environment within the peritoneal cavity of patients with EMs that was similar to tumor-like inflammatory environment. This milieu fosters the implantation, neovascularization, and proliferation of ectopic endometrial tissues, thereby facilitating the progression of EMs. Consistently, this study demonstrated the significant enrichment of pathways associated with the immune system (e.g. antigen processing and presentation, Th17 cell differentiation, Th1 and Th2 cell differentiation, and leukocyte transendothelial migration) and immune diseases (e.g., rheumatoid arthritis and systemic lupus erythematosus) in the EMs group (**Figures 1C, D**). These findings confirm the presence of an immune environment and an inflammation-prone environment in patients with EMs. Consistent with these findings, Chang KK et al. (29) revealed the effect of interleukin-10 (IL-10) and Th17 differentiation on the growth and invasion of endometriotic cells, consequently contributing to the progressive development and aggravation of the EMs. Additionally, several clinical studies have reported that systemic lupus erythematosus and rheumatoid arthritis increase the risk of EMs, suggesting that they have mutual influencing factors with similar immunological and inflammatory responses (5, 30, 31).

In this study, the distribution of most immune cells was dysregulated in EMs, which is consistent with the findings of previous studies (14, 32). In particular, EMs was associated with decreased cytotoxicity of NK cells, an increased abundance and cytotoxicity of macrophages, aberrant activation of T cells and B cells, and enhanced density and number of mast cells and dendritic cells. The activity of NK cells may be suppressed in endometriotic lesions facilitating the evasion of ectopic endometrial cells from immune surveillance and promoting their survival and implantation (33–35). Macrophages are the primary immune cells that produce pro-inflammatory chemokines and are the main source of neurovascularization (36). The disruption of the dynamic equilibrium between M1 and M2 macrophage phenotypes may be involved in the pathogenesis of EMs. Among women with EMs, M2 macrophages are enriched in the peritoneal environment (37, 38). M2 macrophages can exert pathogenic effects by mediating immune suppression, promoting neovascularization and tissue remodeling, and supporting the formation of endometriotic lesions and the fibrotic repair process (39–41). The peritoneal fluid microenvironment in patients with EMs specifically induces the differentiation of monocytes into macrophages rather than dendritic cells (42). This is consistent with the findings of this study. These findings suggest the predominance of macrophages relative to dendritic cells.

This study comprehensively demonstrated that CXCL12, ROBO3, and SCG2 are potential key biomarkers for EMs. The diagnostic values of CXCL12, ROBO3, and SCG2 were established using ROC curve analysis. The levels of CXCL12 in endometriotic lesions were upregulated when compared with those in healthy endometrial tissues, which was consistent with the findings of previous studies. CXCL12, which belongs the CXC chemokine family, is extensively expressed in diverse cells and tissues. CXCL12 effectively binds to CXC motif chemokine receptors 4/7 (CXCR4/7) and subsequently activates multiple signaling pathways (43–45). Additionally, CXCL12 is involved in cell homing, angiogenesis, immune and inflammatory responses, tumor infiltration, and metastasis. Recent studies have reported that CXCL12 and its receptors directly affect the proliferation, migration, invasion, and angiogenesis of endometrial stem cells in normal and ectopic endometrial tissue, promoting the occurrence and progression of endometriotic lesions (46, 47). Additionally, the CXCL12-CXCR4 interaction significantly inhibits the proliferation, migration, and invasion of ectopic endometrial cells (48).CXCL12 is significantly upregulated in endometriotic lesions and enhances the chemotactic activity of bone marrow stem cells (49). These findings are consistent with the results of this study as macrophages and mast cells differentiate from bone marrow stem cells. In this study, the proportions of M1 macrophages, M2 macrophages, and resting mast cells were positively correlated with CXCL12 expression. Consequently, CXCL12 may induce inflammatory reactions by recruiting immune cells to the ectopic lesions, contributing to the formation and development of endometriotic lesions. However, the expression patterns and functional roles of ROBO3 and SCG2 in EMs have not been examined. Limited studies have examined the function of ROBO3, a member of the roundabout guidance receptor (ROBO) family and a transmembrane protein mainly expressed in neuronal cells. The axon guidance ligand (SLIT)/ROBO signaling pathway is reported to play an important role in embryonic development, nervous system development, angiogenesis, and chemotaxis of inflammatory cells (50–52). Denk et al. performed cellular studies and reported that ROBO3 polymorphisms increase the susceptibility to rheumatoid arthritis. Compared with those in healthy individuals, the synovial fibroblast levels of ROBO3, pro-inflammatory cytokines, and cartilage degradation were significantly upregulated in patients with rheumatoid arthritis (53). Recent immunological studies have reported that autoimmune dysfunction may be involved in the development of EMs. Several clinical studies have confirmed that patients with EMs are associated with significantly increased incidence rates of autoimmune diseases, such as systemic lupus erythematosus and rheumatoid arthritis and that disease pathogenesis, clinical symptoms, and disease progression and regression are correlated (30, 31). This study demonstrated that the pathways related to systemic lupus erythematosus, chemokine signaling, and leukocyte transendothelial migration were significantly enriched in the high-ROBO3 expression group. Additionally, the infiltration levels of M2 macrophages and M1 macrophages were upregulated and the infiltration levels of activated dendritic cells, activated NK cells, resting NK cells, and Tregs were downregulated in the high-ROBO3 expression group. ROBO3 was hypothesized to contribute to aberrant immune responses, inflammation, and tissue damage that drive the progression of EMs. Secretoneurin (SN), which is the bioactive peptide product of SCG2 cleavage, is involved in neurotransmitter release, leukocyte migration, and angiogenesis (54, 55). SCG2/SN stimulates ovarian angiogenesis in response to human chorionic gonadotropin via a granulocyte signaling mechanism downstream of the luteinizing hormone receptor (56). In particular, SCG2/SN can prevent endothelial cell apoptosis by activating the vascular endothelial growth factor and MAPK signaling pathways and promote endothelial cell proliferation, migration, and angiogenesis (55, 57, 58), and inducing macrophage accumulation (59). In tumor tissues, SCG2 upregulation may promote the polarization of M0 macrophages to M2 macrophages and their subsequent differentiation into tumor-associated macrophages, enhancing tumor cell invasion and metastasis and angiogenesis and suppressing immune activity (60). In this study, SCG2 was upregulated in EMs tissues. Additionally, cytokine-cytokine receptor interaction, leukocyte transendothelial migration, and MAPK signaling pathway were significantly enriched in the high-SCG2 expression group. SCG2 expression was significantly and positively correlated with the abundances of M2 macrophages and M1 macrophages and negatively correlated with the abundances of activated NK cells and resting NK cells. The upregulation of SCG2 was hypothesized to affect the immune system and promote angiogenesis. These changes impair the ability of the immune system to effectively clear ectopic cells and enable adequate blood supply, promoting the survival and development of ectopic tissues. However, further studies are needed to determine the exact roles of CXCL12, ROBO3, and SCG2 in the pathogenesis of EMs.

Molecular docking analysis examines intermolecular interactions by evaluating the binding energies of small molecules in a network with protein ligands, enabling the mining of potential therapeutic targets. GSEA revealed that steroid hormone biosynthesis was significantly enriched in EMs tissues from the high-CXCL12 and high-ROBO3 expression groups. Dienogest (a novel and highly potent progestin) and goserelin (a gonadotropin-releasing hormone agonist (GnRH-a)) inhibit ovarian steroidogenesis through different mechanisms, downregulate the serum levels of endogenous steroids, and promote ectopic endometrial tissue decidualization, and then atrophy (61).The results of molecular docking analysis revealed that CXCL12, ROBO3, and SCG2 exhibited good binding affinity with dienogest and goserelin, suggesting that they are potential targets for dienogest and goserelin. However, further studies are needed to elucidate the specific regulatory mechanisms.

## Conclusion

This study identified potential diagnostic biomarkers (CXCL12, ROBO3, and SCG2) for EMs and demonstrated the role of immune dysregulation in the EMs pathogenesis. These findings offer valuable insights for developing early diagnostic and targeted therapeutic strategies for EMs.

## Data availability statement

The original contributions presented in the study are included in the article/**Supplementary Material**. Further inquiries can be directed to the corresponding authors.

## Ethics statement

Ethical approval was not required for the studies on humans in accordance with the local legislation and institutional requirements because only commercially available established cell lines were used.

## Author contributions

CZ: Conceptualization, Methodology, Software, Visualization, Writing – original draft. MF: Writing – original draft. YC: Writing – original draft. SL: Writing – original draft. YZ: Software, Visualization, Writing – original draft. JC: Funding acquisition, Software, Visualization, Writing – review & editing. RZ: Conceptualization, Writing – review & editing. XH: Conceptualization, Funding acquisition, Project administration, Writing – review & editing.
