# Supplementary material for: Unraveling immunotherapeutic targets for endometriosis: a transcriptomic and single-cell analysis
Source: Front Immunol. 2023 Nov 16;14:1288263. doi: 10.3389/fimmu.2023.1288263 (PMC10687456; doi:10.3389/fimmu.2023.1288263)
Supplement: Supplementary file 5 [file DataSheet_5.docx]

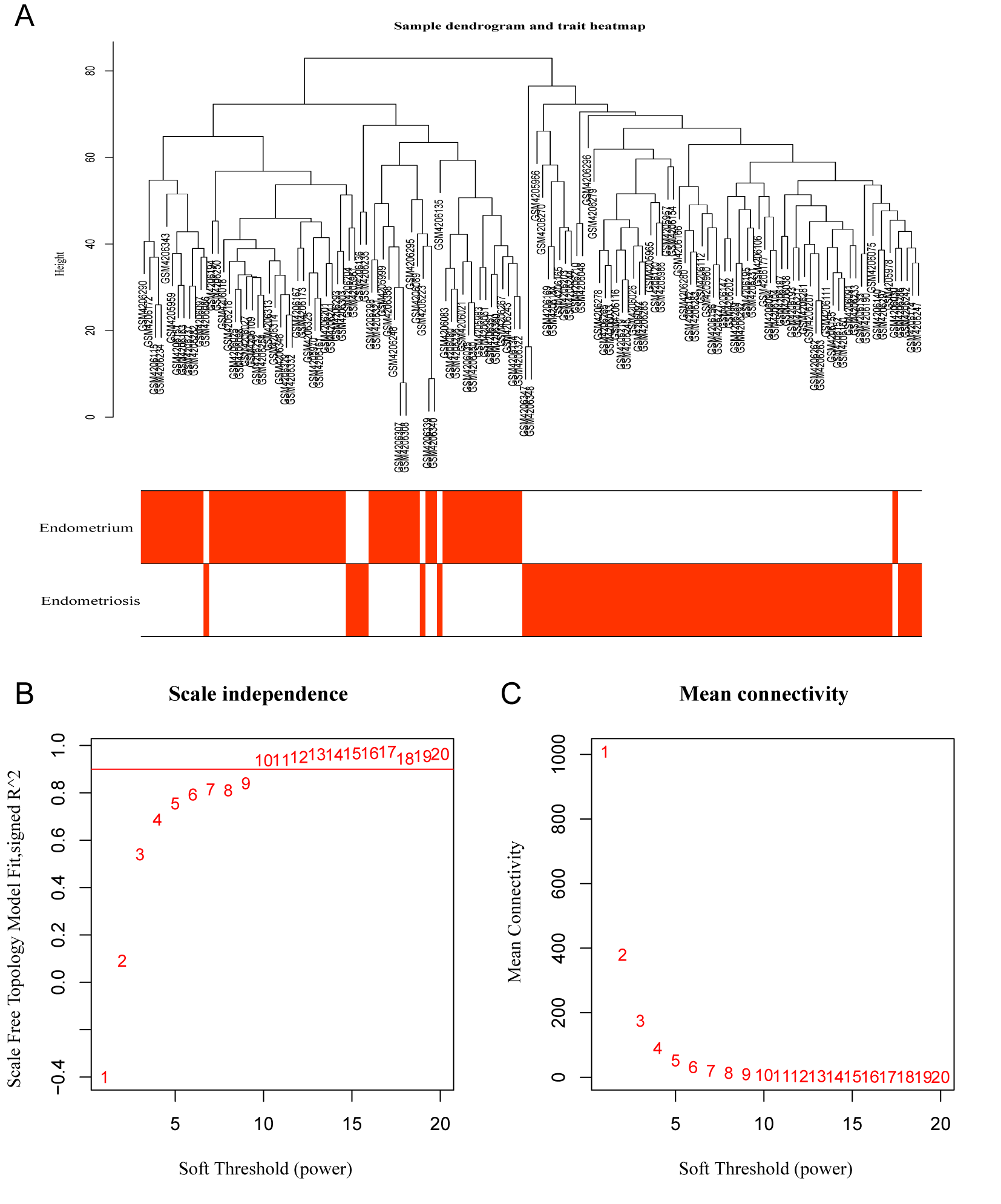


**Supplementary Figure 1** Construction of WGCNA network. **(A)** Sample clustering of GSE141549. **(B)** Soft threshold β = 9 and scale–free topological fit index (R2).


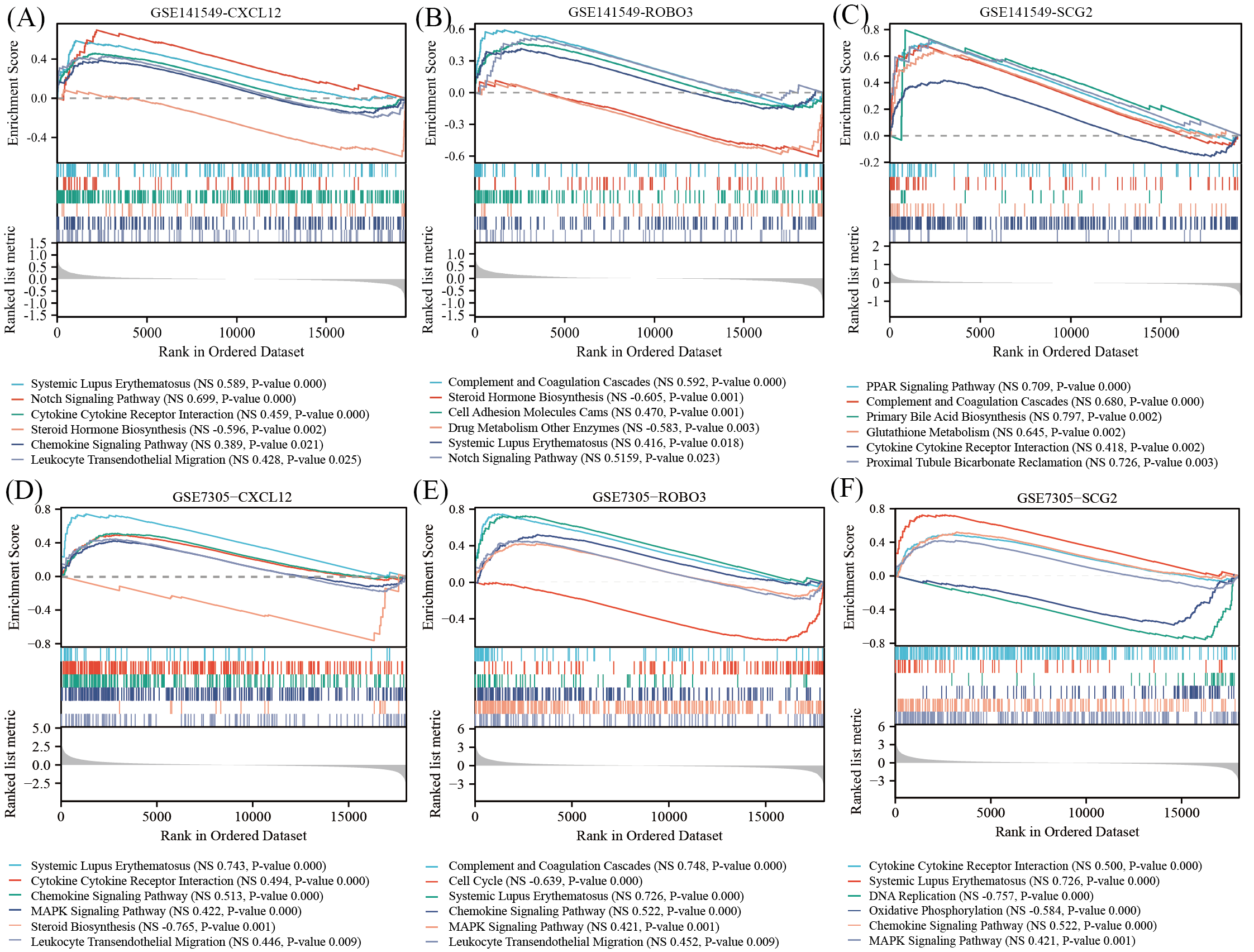


**Supplementary Figure 2** The associated pathway from GSEA analyses for CXCL12 **(A/D)**, ROBO3 **(B/E)**, and SCG2 **(C/F)** (GSE179640/GSE7305).


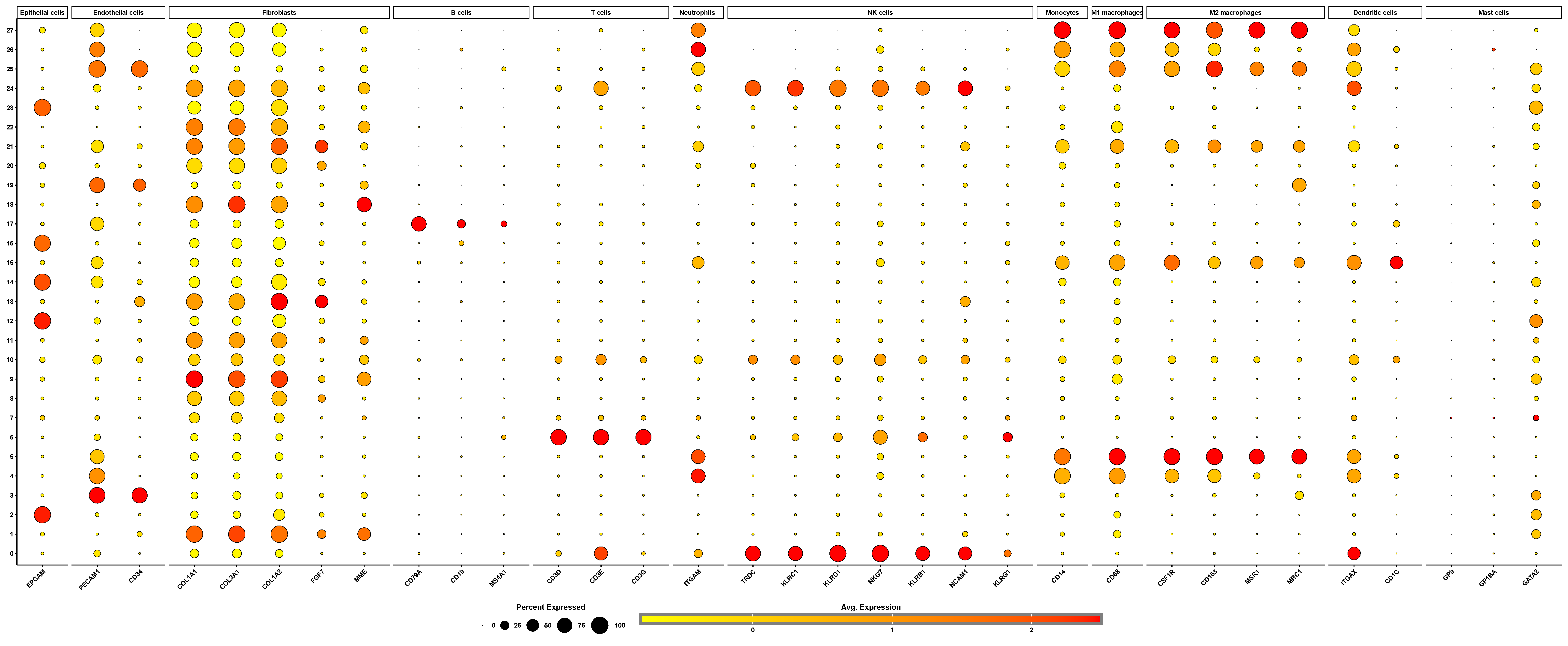

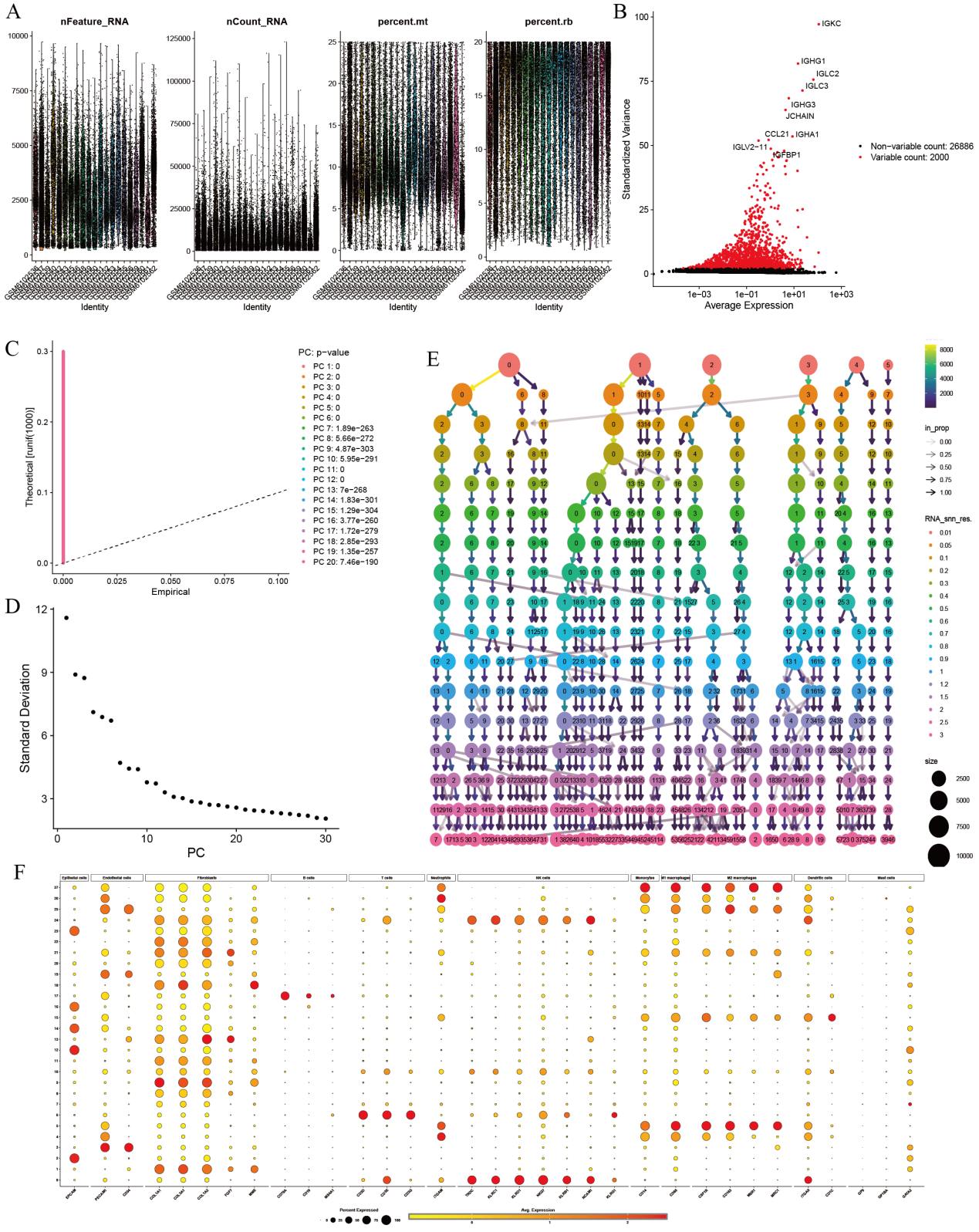


**Supplementary Figure 3** scRNA analysis of endometriosis. **(A)**The genes (features), counts, mitochondrial gene and ribosomal gene percentage of each sample. **(B)** Highly variable genes were colored in red, and the top 10 Highly variable genes were labeled. **(C)** PCs selection using JackStraw function. **(D)** ElbowPlot of PCs. **(E)** Resolution of Seurat clustering results from 0-3.0. **(F)** Expression profiles of the artificially annotated marker genes in each cell cluster.
