# Supplementary material for: Unraveling immunotherapeutic targets for endometriosis: a transcriptomic and single-cell analysis
Source: Front Immunol. 2023 Nov 16;14:1288263. doi: 10.3389/fimmu.2023.1288263 (PMC10687456; doi:10.3389/fimmu.2023.1288263)
Supplement: Supplementary file 6 [file Table_1.docx]

**Supplementary Table 1**

| Gene | Forward primer | Reverse primer |
| --- | --- | --- |
| CXCL12 | 5’-ATTCTCAACACTCCAAACTGTGC-3’ | 5’-ACTTTAGCTTCGGGTCAATGC-3’ |
| ROBO3 | 5’-AACTTGTTCGCGGACTCTCTG-3’ | 5’-TCCTACCCTTGACCCGTTGAG-3’ |
| SCG2 | 5’-TCAACGATGAGATGAAACGCTC-3’ | 5’-TTTGCCCATTCTGTAACCTCC-3’ |
| β-actin | 5’-CCTGGCACCCAGCACAAT-3’ | 5’-GGGCCGGACTCGTCATAC-3’ |
