# Supplementary material for: Unraveling immunotherapeutic targets for endometriosis: a transcriptomic and single-cell analysis
Source: Front Immunol. 2023 Nov 16;14:1288263. doi: 10.3389/fimmu.2023.1288263 (PMC10687456; doi:10.3389/fimmu.2023.1288263)
Supplement: Supplementary file 7 [file Table_2.docx]

**Supplementary Table 2 KEGG functional enrichment analysis of the EMs-related differentially expressed genes**

| Pathway ID | Descrption | KEGG_B_class | Pvalue |
| --- | --- | --- | --- |
| ko05144 | Malaria | Infectious disease: parasitic | 4.66E-07 |
| ko05166 | Human T-cell leukemia virus 1 infection | Infectious disease: viral | 3.19E-06 |
| ko04610 | Complement and coagulation cascades | Immune system | 4.50E-06 |
| ko04514 | Cell adhesion molecules | Signaling molecules and interaction | 1.02E-05 |
| ko04512 | ECM-receptor interaction | Signaling molecules and interaction | 2.26E-05 |
| ko00982 | Drug metabolism - cytochrome P450 | Xenobiotics biodegradation and metabolism | 3.92E-05 |
| ko04612 | Antigen processing and presentation | Immune system | 0.000397 |
| ko04659 | Th17 cell differentiation | Immune system | 0.000796 |
| ko05323 | Rheumatoid arthritis | Immune disease | 0.003025 |
| ko04658 | Th1 and Th2 cell differentiation | Immune system | 0.003599 |
| ko04670 | Leukocyte transendothelial migration | Immune system | 0.003695 |
| ko00980 | Metabolism of xenobiotics by cytochrome P450 | Xenobiotics biodegradation and metabolism | 0.005684 |
| ko04218 | Cellular senescence | Cell growth and death | 0.007425 |
| ko04068 | FoxO signaling pathway | Signal transduction | 0.01234 |
| ko04672 | Intestinal immune network for IgA production | Immune system | 0.013992 |
| ko04115 | p53 signaling pathway | Cell growth and death | 0.015366 |
| ko04110 | Cell cycle | Cell growth and death | 0.018983 |
| ko05322 | Systemic lupus erythematosus | Immune disease | 0.026856 |
| ko04145 | Phagosome | Transport and catabolism | 0.040984 |
| ko04060 | Cytokine-cytokine receptor interaction | Signaling molecules and interaction | 0.048877 |
