# Supplementary material for: Unraveling immunotherapeutic targets for endometriosis: a transcriptomic and single-cell analysis
Source: Front Immunol. 2023 Nov 16;14:1288263. doi: 10.3389/fimmu.2023.1288263 (PMC10687456; doi:10.3389/fimmu.2023.1288263)
Supplement: Supplementary file 8 [file Table_3.docx]

**Supplementary Table 3 GO functional enrichment analysis of the EMs-related differentially expressed genes**

| ID | class | Descrption | Pvalue |
| --- | --- | --- | --- |
| GO:0001568 | Biological Process | blood vessel development | 1.05E-23 |
| GO:0030198 | Biological Process | extracellular matrix organization | 3.30E-21 |
| GO:0042127 | Biological Process | regulation of cell proliferation | 2.34E-21 |
| GO:0001525 | Biological Process | angiogenesis | 2.79E-21 |
| GO:0002682 | Biological Process | regulation of immune system process | 1.03E-19 |
| GO:0002688 | Biological Process | regulation of leukocyte chemotaxis | 4.86E-20 |
| GO:0002684 | Biological Process | positive regulation of immune system process | 7.88E-20 |
| GO:0043069 | Biological Process | negative regulation of programmed cell death | 1.52E-19 |
| GO:0005615 | Cellular Component | extracellular space | 2.39E-19 |
| GO:0005576 | Cellular Component | extracellular region | 4.27E-19 |
| GO:0044421 | Cellular Component | extracellular region part | 6.06E-19 |
| GO:0031012 | Cellular Component | extracellular matrix | 2.94E-18 |
| GO:0005766 | Cellular Component | primary lysosome | 4.25E-18 |
| GO:0035692 | Cellular Component | macrophage migration inhibitory factor receptor complex | 5.09E-18 |
| GO:0042605 | Molecular Function | peptide antigen binding | 6.80E-18 |
| GO:0042379 | Molecular Function | chemokine receptor binding | 7.49E-18 |
| GO:0005125 | Molecular Function | cytokine activity | 1.04E-17 |
| GO:0042608 | Molecular Function | T cell receptor binding | 1.46E-17 |
| GO:0030331 | Molecular Function | estrogen receptor binding | 3.89E-17 |
| GO:0035718 | Molecular Function | macrophage migration inhibitory factor binding | 7.32E-17 |
