# Supplementary material for: Unraveling immunotherapeutic targets for endometriosis: a transcriptomic and single-cell analysis
Source: Front Immunol. 2023 Nov 16;14:1288263. doi: 10.3389/fimmu.2023.1288263 (PMC10687456; doi:10.3389/fimmu.2023.1288263)
Supplement: Supplementary file 9 [file Table_4.docx]

**Supplementary Table 4 The lowest binding energy (kcal/mol) for molecular docking**

| **Drug** | **Target** | | |
| --- | --- | --- | --- |
|  | **CXCL12** | **ROBO3** | **SCG2** |
| **Dienogest** | **-7.77** | **-6.24** | **-5.26** |
| **Goserelin** | **-8.14** | **-6.82** | **-7.15** |
